# Death anxiety predicts fear of progression in people with rheumatic conditions

**DOI:** 10.1111/bjhp.70069

**Published:** 2026-04-09

**Authors:** Bethany Richmond, Louise Sharpe, Jack B. Boyse, Joanne Shaw, Rachel E. Menzies

**Affiliations:** ^1^ School of Psychology, Faculty of Science The University of Sydney Camperdown New South Wales Australia

**Keywords:** anxiety, depression, fear of progression, psychosocial outcomes, quality of life, rheumatic disease

## Abstract

**Background:**

Rheumatic diseases often have a progressive course and place individuals at increased risk of mortality. Despite this, little research has investigated the relationship between death anxiety and fears about disease progression (FoP), and how these might relate to health‐related quality of life (HRQoL) outcomes. This study investigated the relationship between death anxiety, FoP and HRQoL.

**Design:**

A cross‐sectional design with a longitudinal follow‐up at 3 months.

**Methods:**

A total of 145 participants with at least one rheumatic condition were recruited through Prolific. They completed online questionnaires assessing FoP, death anxiety, HRQoL, pain and psychological distress. They also completed an additional measure of FoP 3 months later. A series of regression analyses were conducted to examine whether death anxiety predicted unique variance in FoP cross‐sectionally, as well as three months later. We also investigated whether death anxiety and FoP were associated with HRQoL after controlling for pain, demographics and psychological distress.

**Results:**

Death anxiety contributed unique variance to FoP, even when controlling for other variables of interest, and continued to predict FoP 3 months later. Surprisingly, neither death anxiety nor fear of progression were found to predict unique variance in psychological or physical HRQoL.

**Conclusions:**

These results indicate that death anxiety plays an important role in FoP. As such, death anxiety appears to be a particularly pertinent factor in the experience of FoP for people with rheumatic conditions that deserves further investigation. However, quality of life outcomes may be robust to the impact of death anxiety and FoP.

**Statement of Contribution**

**What is already known on this subject?**

- Rheumatic diseases are progressive conditions that are associated with a higher mortality risk.
- Fear of progression (FoP) is common in rheumatic conditions, yet no effective psychological interventions are available.
- Death anxiety is proposed to contribute to fears about disease recurrence/progression in cancer models.

**What does this study add?**

- Death anxiety uniquely predicted FoP in those with rheumatic conditions.
- Death anxiety and FoP were not found to predict physical or psychological HRQoL after controlling for pain, psychological distress and demographics.
- Highlights the influential role of death anxiety in the experience of FoP in those with rheumatic conditions.

Rheumatic disease refers to a cluster of autoimmune and autoinflammatory conditions that mainly affect the joints and broader musculoskeletal system. There are more than 100 different rheumatic conditions, with some of the most common including rheumatoid arthritis, osteoarthritis, systemic lupus erythematosus, systemic sclerosis and gout (Sangha, 2000). Due to the involvement of the musculoskeletal system, those with rheumatic disease commonly experience pain, joint stiffness and impairments in mobility (Goldblatt & O'Neill, 2013; Sangha, 2000). As rheumatic conditions are presently incurable, treatment focuses on the management of symptoms to minimize the long‐term risk of disability, deformity and increased mortality. For individuals with rheumatic conditions that are auto‐immune in nature, their inflammatory symptoms are the result of dysregulation in the immune system, resulting in it erroneously targeting and damaging healthy cells (Pisetsky, 2023). Rheumatic diseases have highly varied clinical presentations, where the impacts of some are mostly isolated to the musculoskeletal system (e.g., rheumatoid arthritis), but others are more generalized and affect several groups of organs (e.g., systemic lupus erythematosus). The differences in the pattern of organ involvement across different rheumatic diseases, means the course of the conditions can be very unpredictable. As such, understanding the psychosocial impact of these conditions is important.

Research has confirmed heightened rates of depression and anxiety amongst individuals with rheumatic diseases. For example, a meta‐analysis found that one‐quarter of people with Systemic Lupus Erythematosus have a depressive disorder and nearly 40% have an anxiety disorder, according to structured clinical interviews (Zhang et al., 2017). While prevalence estimates vary across studies and conditions, the literature has consistently supported higher rates of depression and anxiety across all rheumatic diseases [e.g., Rheumatoid Arthritis (Matcham et al., 2013); Psoriatic Arthritis (Zusman et al., 2020)]. Alongside these mental health impacts, people with rheumatic disease also face difficulties maintaining employment, with studies finding a lower employment rate relative to the general population (Mau et al., 2005; van der Zee‐Neuen et al., 2017). Poorer quality of life outcomes are also prevalent, including impairments in the social and emotional domains as well as in physical health (Mau et al., 2005). Of particular interest is understanding what might be contributing to these poorer psychosocial outcomes. Relevantly, it has recently been proposed that one of the most common fears of people living with a chronic and unpredictable condition is fear of disease progression (Sharpe, Michalowski, et al., 2023).

Fear of progression (FoP) refers to the fear, concern and worry surrounding the risk of a condition becoming worse, recurring or progressing over time. While there is a paucity of research on FoP in rheumatic disease, the potential importance of FoP to the wellbeing of people with rheumatic diseases is difficult to overstate. In one of the few studies on this topic, Sharpe and colleagues (Sharpe, Richmond, et al., 2023) found that 65% of those with Rheumatoid Arthritis had clinical levels of FoP, meaning that their fears about disease progression were severe and persistent enough that they interfered with daily functioning. Another study of individuals with a range of medical conditions, including cancer, Parkinson's disease, stroke and Multiple Sclerosis, found that FoP was highest amongst people with rheumatic disease (Berg et al., 2011). While it is certainly true that the progression and recurrence of rheumatic disease carry the risk of deformity and disability, as well as interfering levels of pain and fatigue, the fact that FoP is higher in rheumatic diseases than in illnesses such as cancer is still surprising. Currently, there is little research on the impacts of FoP in rheumatic disease. However, there is considerable research on the related construct of fear of cancer recurrence (FCR). FCR has been reported as the most common psychological concern for people living with and beyond cancer with broad impacts on quality of life, mood and functioning (Simard et al., 2013). Aligned with this, a meta‐analysis found that FoP was strongly associated with depression and anxiety across a range of chronic conditions (Sharpe, Michalowski, et al., 2023). Although FoP shares some overlap with general anxiety symptoms, as evidenced by these associations, it is also a distinct construct. Specifically, FoP entails fears about the future deteriorating course of a health condition, which can be distinguished from anxiety disorders where an individual's worry is broader and less specific (e.g., generalized anxiety disorder) or from health anxiety, where the primary fear is developing a serious illness in the future.

The dearth of research on FoP in rheumatic conditions is problematic because, unless we understand the correlates of FoP, our ability to provide treatment is limited. In a randomized controlled trial (RCT) comparing group‐administered Cognitive Behaviour Therapy (CBT) for FoP with an active control in samples with either cancer or arthritis, Herschbach and colleagues (Herschbach et al., 2009) found CBT to be more efficacious than the control treatment for individuals with cancer but not for those with arthritis. Aligned with this, the only other RCT in this area found that a mindfulness‐based stress reduction intervention was more effective for reducing FoP than CBT in those with rheumatoid arthritis (Sharpe, Bisby, et al., 2025). Importantly, these improvements in FoP also mediated improvements in functional outcomes at a 6‐month follow‐up (Sharpe, Menzies, et al., 2025). Although this finding is promising, there remains a paucity of research on treating FoP, especially in other rheumatic diseases. This is concerning as we know that FoP is common and particularly high for people living with rheumatic conditions. Thus, it is crucial to identify factors involved in the aetiology and maintenance of FoP in rheumatic diseases, such that they might inform treatments.

One construct that has been inextricably linked to FCR is death anxiety. Consistent with other anxiety disorders, death anxiety includes an affective (i.e., the fear people experience about death), cognitive (i.e., the unhelpful beliefs about death or the dying process) and behavioural component (i.e., attempts to avoid triggers of death anxiety or death‐related thoughts). Death anxiety has also been proposed to be a transdiagnostic construct that underlies a range of anxiety disorders (Iverach et al., 2014; Menzies et al., 2019). However, it is also distinct from other anxiety disorders due to its focus on mortality and existential fears. Models of FCR highlight how a cancer diagnosis can elicit existential concerns about death anxiety, meaning/purpose and isolation (Fardell et al., 2016; Simonelli et al., 2017). According to Simonelli et al. (Simonelli et al., 2017) when a person is diagnosed with cancer, they immediately fear death which triggers the use of a range of defence mechanisms, such as avoidance, as outlined in existing frameworks, such as terror management theory (TMT) (Goldenberg & Arndt, 2008). The symptoms of a condition and its treatment are appraised as dangerous which gives rise to a range of death‐related cognitions. The removal of these thoughts from explicit awareness is in turn thought to drive the maladaptive cognitive and behavioural processes that trigger FCR and lead to negative psychosocial outcomes (Goldenberg & Arndt, 2008). While this makes sense in the context of cancer, which is a potentially life‐limiting disease, whether these same relationships would be expected in a chronic, but not necessarily life‐limiting set of diseases, is unknown.

However, rheumatological diseases are associated with an increased mortality rate. For instance, individuals with rheumatic diseases are approximately twice as likely as someone of the same age to die of all‐cause mortality (Toledano et al., 2012). While this is an early meta‐analysis and disease‐modifying treatments have improved, more recent disease‐specific meta‐analyses confirmed a heightened risk of mortality for rheumatic diseases such as Vasculitis (Tan et al., 2017) and Systemic Lupus Erythematosus (Restivo et al., 2022). Rheumatoid Arthritis also carries an elevated risk of life‐threatening cardiovascular events, cancer and lung disease (Michaud & Wolfe, 2007). Thus, although death may not be an imminent threat in rheumatic conditions, the possibility of these serious health complications may nevertheless heighten concerns about their future health and lifespan. For those who are already highly fearful of death, they may be more likely to interpret symptoms as being indicative of a deterioration, exacerbating their FoP. If death anxiety does underlie FoP, it could also explain why previous interventions targeting illness‐related concerns have demonstrated limited efficacy in rheumatic populations (Herschbach et al., 2009; Sharpe, Bisby, et al., 2025) and may instead point to the need to address these deeper fears about death to effectively alleviate FoP concerns.

To date, just one study has explored existential concerns in a sample of people with rheumatic conditions. In that study, Sharpe et al. (Sharpe, Richmond, et al., 2023c) explored whether existential concerns, including death anxiety, meaninglessness, guilt, isolation and identity, predict FoP in people with Rheumatoid Arthritis. Stratifying their sample into groups with or without clinical levels of FoP, they found that death anxiety was significantly higher in those in the clinical range. Further, total existential concerns predicted unique variance in FoP, even when controlling for depression, anxiety, pain and disability. Although it is hardly surprising that two anxiety‐based constructs, FoP and death anxiety, are strongly inter‐related in any chronic illness.

Arguably, a better test of the importance of FoP and death anxiety would be to determine their relationship with quality of life. Health‐related quality of life (HRQoL) is an important construct because it aims to determine the degree to which an illness impacts both the physical ability to engage in life activities and the psychological impacts on daily function. Further, HRQoL has mild to moderate correlations with disease activity, including organ damage, in rheumatic and autoimmune conditions such as Systemic Lupus Erythematosus (Shi et al., 2021). Previous research also suggests that FoP is negatively associated with HRQoL outcomes in those with cancer (Simard et al., 2013; Simonelli et al., 2017), lung disease (Liu et al., 2021), diabetes and rheumatic diseases (Herschbach et al., 2005). Comparably, a handful of studies have found that death anxiety is predictive of poorer HRQoL in those with advanced cancer (Sherman et al., 2010), Huntington's disease (Sokol et al., 2023) and interstitial lung disease (Cho & Cho, 2022). If FoP and death anxiety could be shown to be associated with HRQoL in this study, this would strongly support the importance of these constructs in rheumatic disease.

The aim of the current study was to determine the relationship between death anxiety and FoP in people with a range of rheumatological conditions, controlling for other known predictors of FoP. Further, we aimed to determine whether death anxiety and FoP contributed unique variance in the physical and psychological components of HRQoL. Specifically, we hypothesized that:

1. People with clinically significant levels of FoP would score more highly on death anxiety, as well as pain outcomes, psychopathology and HRQoL.
2. Death anxiety affects, maladaptive beliefs and avoidance behaviours would all be significantly associated with FoP.
3. Death anxiety would be associated with unique variance in FoP after controlling for demographic variables, negative emotional states and pain, both in cross‐sectional and prospective analyses.
4. FoP and death anxiety would be independently associated with physical and psychological HRQoL.

## METHODS

### Participants and procedures

Participants were recruited through Prolific, a paid online survey recruitment platform. Eligibility criteria were screened using the Prolific screening system, such that the survey was only visible to those users who indicated that they were over 18 years old and were proficient in English. As prolific does not allow screening for rheumatic disease specifically, eligibility was initially restricted to those with an autoimmune diagnosis. Participants who did not have a rheumatic condition were excluded prior to analyses. Participants self‐selected into the study which was advertised to them on the Prolific platform with a short description of what the study would involve. Upon signing up for the study, participants were given an information statement and consent form. Consent was received from all participants before commencement. Participants who indicated that they had a cancer history were excluded as it is already known that FoP and death anxiety are strongly related in people living with and beyond cancer (Curran et al., 2020) and we wanted to ensure that having cancer survivors in the sample did not artificially inflate the relationships between these two constructs. Researchers are required to report back to Prolific whether a participant's data could ultimately be used in the study and volunteers are only retained when a high proportion of their data is acceptable, resulting in much higher quality data than similar platforms. Participants who took part in this study were re‐contacted through Prolific 3 months after taking part and were asked to complete the WARPS as a measure of FoP again to identify predictors of FoP over time. For completing the study, participants were paid £3.75 which was consistent with Prolific ethical pay guidelines. Ethics approval was obtained by The University of Sydney Human Research Ethics Committee.

### Materials

#### Demographic and disease variables

Participants were administered a series of demographic questions. These collected information about their age, level of education, gender, ethnicity, country of residence and employment. We also asked a series of disease‐related questions to ascertain their specific diagnosis and cancer history.

#### Pain variables

To measure pain, we asked participants if they experienced pain as a symptom of their condition. Those who did were then asked to indicate their current level of pain, how distressing their current pain is and how much pain interferes with their daily life. Responses to these questions were provided on an 11‐point visual analogue scale ranging from 0 to 10. Visual analogue scales (VAS) are routinely used in research to assess pain intensity and have been well validated amongst individuals with chronic pain (Thong et al., 2018) and Rheumatoid Arthritis (Sendlbeck et al., 2015). The inclusion of single items assessing pain interference and pain‐related distress is consistent with the World Health Organization's (WHO) chronic pain classification, which proposes that pain severity should be evaluated across three domains– pain intensity, pain interference and pain‐related distress–and that these domains can be assessed using 11‐point single item rating scales (Korwisi et al., 2021). In support of this, Hay and colleagues (Hay et al., 2022) found that these three single item rating scales demonstrated good construct validity in a chronic pain sample and recommended their use as measures of these pain dimensions.

#### DASS‐21

The Depression Anxiety and Stress Scale‐21 (DASS‐21) (Lovibond & Lovibond, 1996) is a broad measure of psychological distress. Respondents read a series of 21 statements (e.g. ‘I found it difficult to relax’) and indicate how frequently each has applied to them over the past week. Responses are given on a four‐point scale ranging from 0 (‘Never’) to 3 (‘Almost always’). A total score is then calculated for three subscales: depression, anxiety and stress, by summing the scores of the relevant questions and multiplying by two. Our study returned a Cronbach's alpha of .95, indicating strong internal validity.

#### Death anxiety beliefs and Behaviours scale (DABBS)

The DABBS (Menzies et al., 2022) measures the degree to which an individual endorses unhelpful affective responses, beliefs and behaviours related to fears of death and dying. Respondents read 18 statements which each encompass either affect or emotions related to dying (e.g., ‘I am scared of dying’), negative beliefs about death (e.g., ‘My death will be a painful experience’) or avoidance behaviours (e.g., avoiding ‘Thinking about myself dying’). They provide responses on a five‐point scale ranging from 1 to 5, with response anchors varying across the three subscales. A total score for the scale can be calculated by summing all responses, and a total score for each subscale‐affect, beliefs and behaviours. The validity of the DABBS has been demonstrated in both healthy and treatment seeking samples (Menzies et al., 2022). Our study returned a Cronbach's alpha of .95, indicating good internal consistency.

#### Worries about recurrence or progression scale (WARPS)

The WARPS (Sharpe et al., 2024) measures FoP. Participants read a series of 18 statements, including ‘I feel nervous about what the future holds for my illness’, and respond on a five‐point scale ranging from 1 (‘strongly disagree’) to 5 (‘strongly agree’). A total WARPS score ranging from 18 to 90 is calculated by summing the item scores together. The WARPS has been validated in a large sample of people with diabetes, cardiovascular, rheumatic and autoimmune, and respiratory conditions (Sharpe et al., 2024). The current study returned a Cronbach's alpha of .96, indicating good internal consistency. The WARPS has a validated clinical cut‐off of 65 and above that reflects those with clinical levels of FoP (Smith et al., 2024). We conducted Receiver Operating Characteristic (ROC curve) in the original sample. Overall, the area under the curve was excellent (AUC = .895, *n* = 566), and it was acceptable for the rheumatic conditions (AUC = .812). Therefore, we calculated the proportion of individuals with rheumatic disease who scored in the clinical range.

#### The 12‐item short form survey

The 12‐Item Short Form Survey (SF‐12) (Ware Jr. et al., 1996) is a measure of HRQoL. Participants respond to a series of 12 items assessing general health, abilities and emotions, which are then used to calculate an index of physical and psychological HRQoL, a physical component score (PCS) and mental component score (MCS), respectively. Both the PCS and MCS scores were calculated using the algorithm specified by Ware and colleagues (Ware Jr. et al., 1996). This involves the raw item scores being standardized and weighted using US population norms to produce norm‐based t‐scores (*M* = 50, SD = 10). The SF‐12 has been validated in samples with many physical and mental health conditions (Jenkinson et al., 1997; Ware Jr. et al., 1996). Our study returned a Cronbach's alpha of .77 for the PCS and .82 for the MCS, indicating good internal consistency.

### Analyses

All analyses were conducted using SPSS Statistics (Version 28). We wanted to ensure that we had sufficient power to detect medium effect sizes. For our regression analyses, we conducted an a priori sample size using G*Power (Faul et al., 2009) based on hierarchical multiple regression with 8 covariates and 1 independent predictor. To be conservative, we based our estimates on a medium effect of .1, with an alpha of .05 and 90% power to ensure that we could be confident of the results. The power analyses indicated that we needed a minimum sample size of 108. A sample of 112 would provide 90% power to detect medium‐sized correlations (*r* = .30), and 172 participants would be required to detect medium‐sized between‐group differences (*d* = .50) for the independent samples *t*‐tests. To allow for attrition at follow‐up and the exclusion of participants with autoimmune‐only conditions, we aimed to oversample by approximately 20% (*N* = 200) based on the sample needed for the prospective regression analyses. Recruitment was closed once this target was reached. This sample size also ensured that all other analyses were sufficiently powered.

To test hypothesis 1, we split our sample into those scoring below the clinical range of FoP on the WARPS (≤65) and those scoring in the clinical range (≥66). We conducted independent *t*‐tests to examine differences between those with and without clinically significant levels of FoP on a range of psychosocial outcomes. Prior to this, the statistical assumptions necessary for conducting *t*‐tests were examined. This included evaluating the normality of dependent variables using histograms and Q–Q plots. The equality of variances was tested using Levene's test and where variances were unequal (*p* < .05), Welch's *t*‐test was instead reported. To control for multiple comparisons, a Bonferroni correction was applied, where comparisons were considered significant if *p* < .004.

Bivariate correlations were then calculated between FoP (the WARPS) and pain variables, anxiety, depression, physical HRQoL, psychological HRQoL and the three death anxiety (DABBS) subscales (affect, beliefs and behaviours). We decided to take a conservative approach and control for the large number of correlations. Hence, we considered correlations to be significant if *p* < .0007. Hypothesis 2 was assessed by conducting a regression analysis with FoP entered as the dependent variable, and the three subscale scores from the measure of death anxiety entered simultaneously as independent variables.

Our‐third hypothesis was tested using a hierarchical regression with FoP again entered as the dependent variable. To control for demographic variables, age and gender were entered as predictors in the first step. Current pain, pain distress and pain interference were added in the second step to account for the influence of pain‐related factors on FoP. To control for general psychopathology, the depression, anxiety and stress subscales of the DASS‐21 were entered in the third step. To determine whether death anxiety contributed to the unique variance of FoP once all covariates were controlled for, we then entered total DABBS scores in the fourth and final step. We completed these analyses for baseline FoP, as well as FoP 3 months later amongst those who completed both measures (*n* = 104; 72% retention rate).

To test our final hypothesis, we then conducted two separate hierarchical regression equations for the HRQoL subscales (physical and psychological subscales) of the SF‐12. In both cases, we entered participant age, gender, current pain, pain distress, pain interference, depression, anxiety and stress in the first step. This allowed for demographics, pain and general psychopathology to be controlled for prior to entering death anxiety and FoP. Death anxiety and FoP were then entered in the second step to assess their unique contribution to HRQoL outcomes. In the first, physical HRQoL scores were entered as the dependent variable, and in the second, psychological HRQoL scores were entered as the dependent variable.

For each regression analysis, the assumptions of linearity, normality and homoscedasticity of residuals were assessed through visual inspection of residual plots, histograms and Q–Q plots. The Durbin–Watson statistic was used to assess the independence of errors, where values of 1.5–2.5 were considered acceptable. Lastly, multicollinearity was examined using Variance Inflation Factors (VIF), where values of <5 would be considered evidence of high multicollinearity.

## RESULTS

### Preliminary analyses

We recruited 145 people who reported having been diagnosed with a rheumatic disease. The participants had a wide range of rheumatic diseases. Forty‐seven people (32%) had Fibromyalgia, 43 (30%) had Rheumatoid Arthritis, 31 (21%) had Psoriatic Arthritis, 22 (15%) had Osteoarthritis, 12 (8%) had Systemic Lupus Erythematosus, 6 had Chrohn's disease (4%), 5 had Ulcerative Colitis (3%), 4 had Sjögren's Syndrome (3%), 3 had axial spondylitis (2%), 3 had gout (2%), 2 had infectious arthritis (1%) and 1 had scleroderma (1%). Out of the sample, 28.3% (*n* = 41) reported more than one rheumatic diagnosis or an additional non‐rheumatic health condition, such as Hashimoto's disease or chronic fatigue syndrome. The average age of participants was 40.7 years old (SD = 11.80). Most participants were from the United States (*n* = 104, 72%), 24 (17%) and United Kingdom, and the remaining were from Australia, South Africa and Canada (see Table 1). Most of the sample (*n* = 118, 81%) endorsed their ethnicity as white. Thirty‐seven people were male identifying (26%) and 100 (69%) identified as female, with 8 participants indicating they were non‐binary. These proportions are commensurate with the higher proportion of women to men amongst most rheumatic conditions (Oliver & Silman, 2009).

**TABLE 1:** Demographics and descriptives statistics of the total and follow‐up sample.

Variable	Total sample (*N* = 145)	Follow‐up sample (*N* = 104)	Range (min–max)
	Mean (SD)	Mean (SD)	(*N* = 145)
Age	40.65 (11.80)	42.25 (11.18)	20–74
Gender (%)			
Female	100 (69%)	71 (68.3%)	
Male	37 (25.5%)	25 (24%)	
Non‐binary	8 (5.5%)	8 (7.7%)	
Ethnicity			
Asian	6 (4.1%)	3 (2.9%)	
Black or African American	6 (4.1%)	3 (2.9%)	
Hispanic or Latino	4 (2.8%)	3 (2.9%)	
Native American	4 (2.8%)	4 (3.8%)	
White	118 (81.4%)	87 (83.7%)	
Other	7 (4.8%)	4 (3.8%)	
Time since diagnosis (years)	9.50 (7.99)	10.21 (8.43)	1–42
Comorbidities (%)	41 (28.3%)	29 (27.9%)	
Country of residence (%)			
Australia	8 (5.5%)	8 (7.7%)	
Canada	7 (4.8%)	7 (6.7%)	
South Africa	2 (1.4%)	2 (1.9%)	
United Kingdom	24 (16.6%)	2 (1.9%)	
United States	104 (71.7%)	22 (21.2%)	
Education (%)			
High school	25 (17.2%)	25 (17.2%)	
Trade certificate	25 (17.2%)	25 (17.2%)	
Undergraduate degree	39 (26.9%)	39 (26.9%)	
Postgraduate degree	15 (10.3%)	15 (10.3%)	
Employment status (%)			
Unemployed	26 (17.9%)	15 (14.4%)	
Student	6 (4.1%)	5 (4.8%)	
Casual	3 (2.1%)	2 (1.9%)	
Part‐time	24 (16.6%)	19 (18.3%)	
Full‐time	65 (44.8%)	48 (46.2%)	
Homemaker	14 (9.7%)	11 (10.6%)	
Retired	7 (4.8%)	4 (3.8%)	
Pain intensity	4.44 (2.06)	4.59 (2.14)	1–10
Pain distress	3.92 (2.47)	3.96 (2.59)	0–10
Pain interference	5.77 (2.74)	5.75 (2.77)	0–10
PCS‐12	35.79 (9.78)	36.40 (10.28)	15.57–61.72
MCS‐12	39.22 (11.80)	38.67 (11.58)	17.58–62.54
DABBS behaviours	18.55 (7.59)	18.27 (7.66)	7–35
DABBS affect	12.61 (5.18)	12.53 (5.08)	4–20
DABBS beliefs	21.14 (6.96)	21.74 (6.79)	7–35
DABBS total	52.31 (16.25)	52.54 (16.33)	18–90
DASS‐21 depression	15.99 (12.28)	16.73 (12.63)	0–42
DASS‐21 anxiety	12.36 (9.08)	12.29 (9.34)	0–42
DASS‐21 stress	16.94 (9.90)	17.02 (9.87)	0–42
WARPS	71.69 (11.81)	72.63 (11.32)	34–90

*Note*: Pain variables were assessed using 11‐point visual analogue scales (range 0–10). Ranges reported in the table are the observed minimum and maximum values in the total sample.

Abbreviations: DABBS, Death Anxiety Beliefs and Behaviours Scale (Range Affect 4–20; Beliefs 7–35, Behaviours 7–35; Total 18–90); DASS‐21, Depression Anxiety Stress Scales‐21 (subscales range 0–42); MCS, Mental Component Score (range 0–100); PCS, Physical Component Score (range 0–100); WARPS = Worries about Recurrence or Progression Scale (range 18–90).

### Comparison of those with and without clinical levels of FoP

Results demonstrated that 106 participants scored in the clinically significant range (73%). Assumption checks revealed that the outcomes of interest were mostly approximately normally distributed, with some measures showing a mild to moderate positive skew (e.g., anxiety and death anxiety affect). As this is typical of psychosocial data and parametric tests are robust, no transformations were applied. As can be seen from Table 2, those with clinically significant levels of FoP had higher levels of pain intensity [*t*(142) = 3.83, *p* < .001], pain interference [*t*(142) = 4.18, *p* < .001] and pain‐related distress [*t*(142) = 3.51, *p* < .001]. Those with clinically significant levels of FoP also had higher levels of depression [*t*(88.6) = 3.59, *p* < .001] and anxiety [*t*(99.6) = 4.40, *p* < .001] and had poorer physical [*t*(54.2) = −3.41, *p* = .001] HRQoL. However, the groups did not differ on stress [*t*(143) = 2.52, *p* = .013] or psychological HRQoL [*t*(143) = −2.92, *p* = .004]. Importantly, death anxiety also differed between those with and without clinically significant levels of FoP [*t*(143) = 4.44, *p* < .001], and this was also evident for two of the three subscales: affect (*t*(143) = 3.91, *p* < .001) and beliefs [*t*(143) = 4.49, *p* < .001], but not for behaviours [*t*(143) = 2.58, *p* = .011].

**TABLE 2:** Differences between participants with clinical and non‐clinical levels of fear of progression.

Variable	Clinical group	Non‐clinical group	*t*	*p*	Cohen's *d*
	Mean (SD)	Mean (SD)			
Pain intensity	4.82 (1.99)	3.39 (1.93)	3.83	**<.0005**	.72
Pain interference	6.31 (2.58)	4.26 (2.64)	4.18	**<.0005**	.79
Pain distress	4.34 (2.45)	2.76 (2.16)	3.51	**<.0005**	.66
DASS‐21 depression	17.90 (12.63)	10.82 (9.62)	3.59	**< .001**	.59
DASS‐21 anxiety	13.98 (9.40)	7.95 (6.40)	4.40	**<.0005**	.69
DASS‐21 stress	18.17 (10.19)	13.59 (8.27)	2.52	.013	.47
PCS‐12	33.95 (8.45)	40.77 (11.39)	−3.41	**<.0005**	−.73
MCS‐12	37.52 (11.57)	43.82 (11.32)	−2.92	.004	−.55
DABBS total	55.73 (15.87)	43.03 (13.55)	4.44	**<.0005**	.83
DABBS affect	13.58 (4.93)	9.97 (4.97)	3.91	**<.0005**	.73
DABBS beliefs	22.62 (6.76)	17.13 (5.86)	4.49	**<.0005**	.84
DABBS behaviours	19.52 (7.47)	15.92 (7.39)	2.58	.011	.48

*Note*: Bolded *p* values indicate *p* values that are below the level of significance *p* = 0.004 (Bonferroni correction).

Abbreviations: DABBS, Death Anxiety Beliefs and Behaviours Scale (Range Affect 4–20; Beliefs 7–35, Behaviours 7–35; Total 18–90); DASS‐21, Depression Anxiety Stress Scales‐21 (Range subscales 0–42); MCS, Mental Component Score (Range 0–100); PCS, Physical Component Score (Range 0–100).

### Correlational analyses

Table 3 presents the intercorrelations between variables. As expected, FoP was positively correlated with all pain variables, depression, anxiety, stress and the death anxiety affect and beliefs subscales. However, it did not correlate significantly with the death anxiety behaviours subscale (*r* = .24, *p* = .003). Psychological and physical HRQoL were negatively associated with all pain variables. Psychological, but not physical HRQoL, was also negatively correlated with FoP, all subscales of the DASS‐21 and the beliefs death anxiety subscale. In contrast, physical HRQoL was not associated with FoP (*p* = .003), nor any of the death anxiety (*p*s ≥ .402) or DASS‐21 subscales (*p*s ≥ .004). Similarly, psychological HRQoL was not significantly correlated with the behaviours or affect death anxiety subscales (*p*s ≥ .004).

**TABLE 3:** Pearson correlations between FoP and psychosocial variables.

	Pain intensity	Pain distress	Pain interference	WARPS	PCS	MCS	DABBS behaviour	DABBS affect	DABBS beliefs	DASS‐21 depression	DASS‐21 anxiety	DASS‐21 stress
Pain intensity	–	.80*	.69*	.43*	−.45**	−.29*	.15	.07	.22	.31*	.24	.14
Pain distress		–	.61*	.47*	−.35*	−.34*	.16	.15	.26	.38*	.35*	.23
Pain interference			–	.42*	−.59*	−.46*	.24	.07	.11	.42*	.40*	.27
WARPS				–	−.24	−.39*	.24	.40*	.43*	.44*	.41*	.38*
PCS‐12					–	.06	−.08	.09	−.02	−.10	−.24	−.12
MCS‐12						–	−.19	−.18	−.33*	−.78*	−.60*	−.65*
DABBS behaviours							–	.43*	.48*	.10	.26	.12
DABBS affect								–	.66*	.20	.26	.30*
DABBS beliefs									–	.38*	.48*	.44*
DASS‐21 Depression										–	.66*	.73*
DASS‐21 anxiety											–	.72*
DASS‐21 stress												–

*Note*: *p < .0007.

Abbreviations: DABBS, Death Anxiety Beliefs and Behaviours Scale (range affect 4–20; Beliefs 7–35, Behaviours 7–35; Total 18–90); DASS‐21, Depression Anxiety Stress Scales‐21 (Range subscales 0–42); FoP, fear of progression; MCS, Mental Component Score (Range 0–100); PCS, Physical Component Score (Range 0–100); WARPS, Worries about Recurrence or Progression Scale (Range 18–90).

### Regression analyses

To identify which aspects of death anxiety most strongly contributed to FoP, we conducted a multiple regression analysis to predict FoP. We entered death anxiety affect, beliefs and behaviours as independent variables. The overall model was significant and accounted for 20.9% of the variance in FoP, *F*(3,141) = 12.42, *p* < .001 (see Table 4). Affect was a significant predictor of FoP, *b* = .48, SE = .23, *t*(141) = 2.06, *p* = .041, as were beliefs, *b* = .48, SE = .18, *t*(141) = 2.71, *p* = .008. However, behaviours were not found to predict FoP. The residual plots, histograms and Q–Q plots indicated that the assumptions of linearity, normality and homoscedasticity were met. The Durbin‐Watson statistic was 2.08 and VIF values were all within acceptable limits (<2).

**TABLE 4:** Simultaneous death anxiety subscales on fear of progression.

	*B*	SE	β	*t*	*p*
Death anxiety behaviours	.03	.13	.02	.21	.831
Death anxiety affects	.48	.23	.21	2.06	.**041**
Death anxiety beliefs	.48	.18	.28	2.71	.**008**

*Note*: Bolded *p* values indicate *p* values that are below the level of significance *p* = 0.004 (Bonferroni correction).

To determine whether death anxiety contributed to FoP over other psychosocial outcomes, we conducted a hierarchical multiple regression (see Table 5). When adding age and gender as predictors of FoP in step one, the overall model explained 1% of the variance in FoP, which was not a significant contribution, *F*(2,141) = .70, *p* = .496. Neither age nor gender was a significant predictor of FoP. In step 2, adding current pain, pain distress and pain interference explained an additional 26.6% of variance, which was a significant contribution, *F*(3,138) = 16.93, *p* < .001. However, only pain distress contributed individually to the model, *b* = 1.62, SE = .58, *t*(138) = 2.81, *p* = .006, but pain distress did not remain a significant predictor in the final step of the model. In the third step, adding depression, anxiety and stress to the model explained an additional 6.4% of variance, which was a significant contribution, *F*(3,135) = 4.33, *p* = .006. However, none of the added variables were found to significantly predict FoP. When death anxiety was added to the model, it accounted for a small but significant 7.5% of variance *F*(1,134) = 17.06, *p* < .001. In addition to death anxiety, *b* = .22, SE = .06, *t*(134) = 4.13, *p* < .001, gender was also a significant predictor of FoP, *b* = 3.43, SE = 1.57, *t*(134) = 2.19, *p* = .030. Visual inspection of residual plots, histograms and Q–Q plots confirmed that all assumptions were met. The Durbin–Watson statistic was 2.1 and although some VIFs were moderately elevated, this was within the accepted range (VIFs <4).

**TABLE 5:** Hierarchical multiple linear regression controlling for demographics, pain and negative emotional states on the relationship between death anxiety and fear of progression.

Variable	Step 1	Step 2	Step 3	Step 4
	B (SE)			
Age	−.05 (.08) *p* = .562	−.14 (.07) *p* = .068	−.04 (.08) *p* = .588	−.03 (.07) *p* = .707
Gender	1.98 (1.88) *p* = .295	1.97 (1.65) *p* = .234	2.30 (1.64) *p* = .162	3.43 (1.57) ***p* = .030**
Pain intensity	–	.38 (.78) *p* = .629	.64 (.76) *p* = .400	.43 (.72) *p* = .549
Pain distress	–	1.62 (.58) ***p* = .006**	1.15 (.58) ***p* = .047**	1.03 (.54) *p* = .060
Pain interference	–	.73 (.43) *p* = .097	.29 (.44) *p* = .507	.38 (.42) *p* = .370
depression	–	–	.12 (.11) *p* = .274	.16 (.10) *p* = .133
Anxiety	–	–	.12 (.14) *p* = .399	−.04 (.14) *p* = .977
Stress	–	–	.14 (.14) *p* = .347	.07 (.14) *p* = .589
Death anxiety	–	–	–	.22 (.06) ***p* < .001**
*R*	.099 *p* = .496	.526 *p* < .001	.583 *p* < .001	.644 *p* < .001
*R* ^2^ ∆	−.004 *p* = 496	.266 *p* < .001	.064 *p* = .006	.075 *p* < .001

*Note*: Bolded *p* values indicate *p* values that are below the level of significance *p* = 0.004 (Bonferroni correction).

To determine whether death anxiety remained a predictor of FoP 3 months later, we repeated the hierarchical regression with FoP score at 3 months (See Table 6). Gender and age failed to predict FoP 3 months later *F*(2,100) =1.12, *p* = .329. However, adding the pain variables on the second step contributed an additional 27.5% to the variance in FoP, *F*(3, 97) = 12.64, *p* < .001. Interestingly, it was again pain distress that predicted FoP, *b* = 1.81, SE = .71, *t*(97) =2.56, *p* = .012. However, pain interference was also a significant predictor of FoP, *b* = 1.49, SE = .56, *t*(97) = 2.65, *p* = .009. However, as in the cross‐sectional relationships, pain distress and pain interference did not remain a predictor in subsequent steps of the equation. Adding stress, depression and anxiety on the third step again accounted for additional variance (9.5%) which was significant, *F*(3,94) = 4.91, *p* = .003. Adding death anxiety to the final step contributed another 6.9% to the variance *F*(1,93) = 11.90, *p* < .001. In contrast to the cross‐sectional results, depression also predicted subsequent FoP, *b* = .31, SE = .12, *t*(93) = 2.51, *p* = .014 in this final step. Gender was also found to emerge as a significant predictor of FoP, *b* = 4.11, SE = 1.93, *t*(93) = 2.13, *p* = .036. All assumptions were confirmed through examination of residual plots, histograms and Q–Q plots. The Durbin–Watson statistic was 2.2 and the VIF values suggested an acceptable amount of multicollinearity (<4.2).

**TABLE 6:** Hierarchical multiple linear regression controlling for demographics, pain and negative emotional states on the relationship between death anxiety and fear of progression at follow‐up.

Variable	Step 1	Step 2	Step 3	Step 4
	B (SE)			
Age	−.14 (.11) *p* = .202	−.19 (.10) *p* = .054	−.04 (.10) *p* = .710	.01 (.10) *p* = .907
Gender	1.46 (2.22) *p* = .513	.91 (1.94) *p* = .641	2.00 (1.94) *p* = .305	4.11 (1.93) ***p* = .036**
Pain intensity	–	−1.00 (.94) *p* = .287	−.78 (.91) *p* = .397	−1.23 (.87) *p* = .163
Pain distress	–	1.81 (.71) ***p* = .012**	1.18 (.69) *p* = .091	1.11 (.66) *p* = .094
Pain interference	–	1.49 (.56) ***p* = .009**	.93 (.56) *p* = .103	1.00 (.53) *p* = .065
Depression	–	–	.31 (.12) ***p* = .014**	.40 (.12) ***p* = .001**
Anxiety	–	–	.09 (.17) *p* = .616	−.04 (.17) *p* = .797
Stress	–	–	.03 (.18) *p* = .875	−.07 (.18) *p* = .687
Death anxiety	–	–	–	.23 (.18) ***p* < .001**
*R*	.148 *p* = .329	.545 *p* < .001	.626 *p* < .001	.679 *p* < .001
*R* ^2^ ∆	.002 *p* = .329	.275 *p* < .001	.095 *p* = .003	.069 *p* < .001

*Note*: Bolded *p* values indicate *p* values that are below the level of significance *p* = 0.004 (Bonferroni correction).

In our final analyses, we conducted two hierarchical regression analyses to predict the physical and psychological components of HRQoL. Age, gender, current pain, pain distress, pain interference, depression, anxiety and stress were added in the first step and explained 42.1% of the variance in physical HRQoL, which was significant, *F*(8,135) = 12.27, *p* < .001 (see Table 7). Of these, pain interference, *b* = −2.10, SE = .34, *t*(135) = −6.13, *p* < .001, and depression, *b* = .27, SE = .09, *t*(135) = 3.14, *p* = .002 were significant predictors of physical HRQoL and remained significant predictors in the final step. Adding in death anxiety and FoP in the second step only explained an additional .9% of variance, which was not a significant contribution, *F*(2,133) = 1.07, *p* = .35. Death anxiety and FoP did not significantly predict physical HRQoL. Assumption checks indicated that linearity, normality and homoscedasticity were met. The independence of residuals was acceptable with a Durbin–Watson statistic of 1.8. However, the VIF values were moderately elevated, with the DASS‐21 subscales and pain measures ranging from 2.6 to 3.7. Although this was within accepted limits, when examining the results, it was noted that the direction of the relationship between depression and physical HRQoL (*b* = .27) was inconsistent with the correlation found (*r* = −.10). This indicates that multicollinearity may have caused a suppression effect. As a result, this coefficient should be considered uninterpretable.

**TABLE 7:** Hierarchical multiple linear regression of variables on physical health‐related quality of life.

Variable	Step 1	Step 2
	B (SE)	
Age	−.01 (.06) *p* = .920	−.03 (.06) *p* = .967
Gender	−1.32 (1.27) *p* = .299	−.93 (1.31) *p* = .480
Pain intensity	−.76 (.59) *p* = .200	−.81 (.59) *p* = .173
Pain distress	.35 (.45) *p* = .434	.34 (.45) *p* = .457
Pain interference	−2.10 (.34) ***p* < .001**	−2.06 (.34) ***p* < .001**
Depression	.27 (.09) ***p* = .002**	.28 (.09) ***p* = .001**
Anxiety	−.15 (.11) *p* = .176	−.19 (.11) *p* = .103
Stress	−.09 (.11) *p* = .405	−.11 (.11) *p* = .332
Death anxiety	–	.07 (.05) *p* = .150
Fear of progression	–	−.02 (.07) *p* = .767
*R*	.649 *p* ≤ .001	.656 *p* < .001
*R* ^2^ ∆	.421 *p* ≤ .001	.009 *p* = .347

*Note*: Bolded *p* values indicate *p* values that are below the level of significance *p* = 0.004 (Bonferroni correction).

The same hierarchical regression was repeated to determine relationships with psychological HRQoL (See Table 8). The first step explained 65.4% of the variance in psychological HRQoL, *F*(8,135) = 31.86, *p* < .001, which was significant. Again, pain interference, *b* = −.89, SE = .32, *t(*135) = −2.74, *p* = .007, and depression, *b* = −.57, SE = .08, *t*(135) = −7.13, *p* < .001, were significant predictors of psychological HRQoL and remained significant predictors in the final step. When death anxiety and FoP were added in the second step, they only explained an additional .2% of variance in psychological HRQoL, which was not significant, *F*(2,133) = .36, *p* = .700. Neither death anxiety nor FoP were significant predictors of psychological HRQoL. Visual inspection of residual plots, histograms and Q–Q plots confirmed the assumptions of linearity, normality and homoscedasticity. The Durbin–Watson statistic was 1.9 and multicollinearity was acceptable according to the VIF values (VIFs <4).

**TABLE 8:** Hierarchical multiple linear regression of variables on psychological quality of life.

Variables	Step 1	Step 2
	B (SE)	
Age	−.08 (.06) *p* = .159	−.08 (.06) *p* = .151
Gender	.14 (1.20) *p* = .906	−.05 (1.24) *p* = .966
Pain intensity	.42 (.56) *p* = .453	.45 (.56) *p* = .424
Pain distress	.08 (.42) *p* = .854	.09 (.43) *p* = .831
Pain interference	−.89 (.32) ***p* = .007**	−.90 (.33) ***p* = .007**
Depression	−.57 (.08) ***p <* .001**	−.58 (.08) ***p <* .001**
Anxiety	−.04 (.11) *p* = .681	−.02 (.11) *p* = .829
Stress	−.21 (.11) *p* = .053	−.20 (.11) *p* = .069
Death anxiety	–	−.04 (.04) *p* = .416
Fear of progression	–	.01 (.07) *p* = .946
*R*	.809 *p* ≤ .001	.810 *p* < .001
*R* ^2^ ∆	.654 *p* ≤ .001	.002 *p* = .700

*Note*: Bolded *p* values indicate *p* values that are below the level of significance *p* = 0.004 (Bonferroni correction).

### Sensitivity analysis

Given that there was a 31% dropout in the number of participants who completed the 3‐month follow‐up measure, we conducted a sensitivity analysis to determine whether there were any differences in demographic characteristics and baseline outcomes between those who completed the follow‐up and those who did not. Completers were found to be older on average than non‐completers, *t*(143) = −2.66, *p* = .009 (See Table 1). There was no significant difference between the two groups on any baseline outcomes, including pain measures, HRQoL, FoP, death anxiety, depression, anxiety or stress (*p*s ≥ .101) (see Table 1).

## DISCUSSION

This study aimed to investigate the relationship between death anxiety and FoP and to determine whether death anxiety and FoP are associated with physical and psychological HRQoL. Our results confirmed that severe levels of FoP are common amongst those with rheumatic conditions, with 73% of the participants scoring in the clinical range. Those with clinically significant FoP had more pain, psychopathology and poorer physical HRQoL, as well as higher levels of death anxiety. Further, we found that death anxiety alone accounted for 20% of the variance in FoP. Specifically, it was death anxiety‐related affect and maladaptive beliefs, rather than avoidance behaviours, that were associated with FoP. Indeed, death anxiety continued to account for individual variance in FoP even when controlling for demographic variables, as well as psychopathology and pain outcomes. Importantly, death anxiety also predicted FoP 3 months later, indicating that for people who have high levels of death anxiety, FoP remains higher 3 months later. Surprisingly, neither FoP nor death anxiety accounted for unique variance over and above other predictors in either psychological or physical aspects of HRQoL. Taken together, these results confirm an important contribution for death anxiety in FoP for those with rheumatic disease.

It is worth noting that the very high rates of clinical FoP found in this study (73%) were more than three times higher than the clinical rates found in adults with diabetes (23%) (Wang et al., 2022) and cancer (19%) (Luigjes‐Huizer et al., 2022). However, it is in keeping, if slightly higher, than what has been reported previously in adults with Rheumatoid Arthritis (65%) (Sharpe, Richmond, et al., 2023), indicating that rheumatic disease might be particularly susceptible to fear of disease progression. Although it is possible that the higher clinical rates found in this study are the result of differences in the measure used to identify clinical cases, as Sharpe and colleagues used the Fear of Progression Questionnaire (FOPQ) (Herschbach et al., 2005) rather than the WARPS. The WARPS was validated against the FOPQ short‐form and so this explanation is unlikely. Nevertheless, this finding in conjunction with our results robustly demonstrates the very high rates of clinical FoP in those with rheumatic conditions.

In the mental health literature, death anxiety has been argued to be a transdiagnostic construct that contributes to the revolving door of mental health problems (Iverach et al., 2014; Menzies et al., 2024). Specifically, death anxiety is argued to underlie a range of mental health conditions and has been shown to be associated with the severity of psychopathology across a range of mental health conditions (Menzies et al., 2019). Therefore, if one conceptualizes FoP as an illness‐specific form of anxiety, the strong relationship between death anxiety and FoP is hardly surprising. Nevertheless, by constructing a hierarchical regression and controlling for a range of pain‐related outcomes known to be associated with FoP and psychopathology (i.e., depression, anxiety and stress), we provided a stringent test as to whether FoP and death anxiety have a unique relationship that cannot be accounted for by other known predictors of both constructs. Furthermore, the fact that death anxiety predicted FoP 3 months later indicates that fears of death are important to persistent FoP.

Our results were also able to characterize the aspects of death anxiety that are associated with FoP. Simonelli et al. (2017) argued that, in the context of FCR, death anxiety led to changes in both emotional and cognitive processing that contributed to FCR. Indeed, it was the emotional (affect) and cognitive (beliefs) aspects of death anxiety, rather than the behavioural aspects, that were associated with FoP. Items on the behaviour scale relate to avoiding activities that contain reminders of death, such as media stories about death and dying, reading a novel or watching a TV show about someone who is dying (Menzies et al., 2022). It may be that in the context of chronic illness, the trigger to become worried about death anxiety is internal rather than external. That is, if one has a chronic condition that is known to be associated with increased mortality, then it may be the presence of symptoms or illness‐related triggers that are avoided. As such, the behaviour subscale may have less relevance to FoP.

Surprisingly, FoP and death anxiety did not predict the psychological or physical components of HRQoL. The mental composite score of the SF‐12 correlates highly with psychopathology. For example, in people with rheumatoid arthritis, those who were depressed had poorer HRQoL on all aspects of the scale (Zhang et al., 2020). Indeed, in the present study, pain interference and depression strongly contributed to the psychological aspects of HRQoL. It is possible that, as the SF‐12 broadly assesses psychological and physical health, it was not sensitive to the impact of existential and illness‐related concerns. It could also be that death anxiety and FoP are not associated with direct impacts on psychological or physical functioning broadly and instead that their role is greater in shaping healthcare behaviours. For example, in the cancer literature, FCR has been found to predict greater healthcare use (Simard et al., 2013; Thewes et al., 2012; Williams et al., 2021), such as unplanned visits to the GP, which could reflect excessive reassurance seeking. Considering this, it is recommended that future studies investigate how death anxiety and FoP may influence healthcare use and related behaviours in the rheumatic population.

Notably, in a range of other conditions, pain intensity has been a strong predictor of both high FoP (Michalski et al., 2024; Pradhan et al., 2021, 2022) and poor HRQoL (Gore et al., 2005; Shrestha et al., 2024; Szymczak et al., 2025). In the present study, pain interference was unsurprisingly associated with both psychological and physical HRQoL; however, pain intensity was not uniquely associated with either HRQoL domain. Moreover, pain intensity, interference and distress were not independently predictive of FoP either cross‐sectionally or prospectively. This differs from findings in other chronic illnesses. For example, pain severity has been shown to be associated with FoP in people with diabetes (Michalski et al., 2024), ovarian (Pradhan et al., 2021) and breast cancer (Pradhan et al., 2022). However, it is consistent with the only other study to look at the relationship between pain and FoP in rheumatic conditions, which likewise found that neither pain interference nor intensity were independently predictive of FoP (Boyse et al., 2024). Instead, chronic pain acceptance was found to predict FoP over and above the other pain covariates. This suggests that, as opposed to the intensity of pain or its degree of interference, it may be a lack of acceptance of pain that confers the most vulnerability to FoP. An alternative explanation proposed by Boyse and colleagues (Boyse et al., 2024) was that other symptoms of disease activity, such as inflammation, stiffness, warmth and fatigue may be more concerning and indicative of a progression—particularly later in the disease course where pain has become chronic.

### Strengths and clinical implications

This study featured several important strengths, which are worth discussing. Firstly, this study adds to the growing literature that has found very high rates of clinical FoP in people with rheumatic disease. As more than 70% of participants scored above the FoP clinical cut‐off, this result emphasizes the urgency of addressing FoP in this population, especially as presently there is a lack of effective interventions to support them. To aid with this, hierarchical regression analyses were used to isolate and stringently test the contribution of death anxiety to FoP, after accounting for other psychological covariates. Moreover, the implementation of a prospective design, whereby FoP was measured again at a 3‐month follow‐up, was another notable strength of this study's design. Together, these strengths add further weight to the finding that death anxiety contributes to FoP.

Importantly, as the first study to examine the role of death anxiety in FoP, our results also provide some initial directions for how FoP may begin to be addressed. Firstly, they suggest that individuals presenting with high FoP should also be screened for death anxiety. The DABBS (Menzies et al., 2022) would be an acceptable screener as it has been found to reliably identify individuals with clinical levels of death anxiety. Moreover, given that a previous RCT found that CBT interventions focused specifically on fears of progression were less effective than mindfulness for treating FoP (Sharpe, Bisby, et al., 2025), one potential pathway for increasing treatment efficacy could be to also address individuals' fears about death. Promisingly, there are effective treatments which target death anxiety (Menzies et al., 2018) that could be incorporated into FoP interventions. The most effective interventions for death anxiety employ CBT techniques which restructure unhelpful cognitions and beliefs about death, reduce avoidance behaviours and facilitate graded exposure to death‐related triggers (Menzies et al., 2018). In addition to establishing death anxiety as a contributor to FoP, the present study also identified the specific role of death anxiety affect and beliefs in FoP. Taking these findings into account, interventions for FoP may be more effective if they include treatment components designed to target unhelpful beliefs about death and how this might relate to their illness progression. However, as discussed earlier, avoidance of death‐related triggers, as measured by the behaviours subscale of the DABBS, appeared to be less relevant to FoP in this population. This raises the question of whether traditional exposure‐based approaches utilizing external death‐related triggers would be effective in this context. Instead, it may be more useful to use an illness‐specific version of exposure, such as the worst‐case scenario that has shown some early promise in the FCR literature (Arch et al., 2024; Moran et al., 2017).

### Limitations

It should be noted that the present study had a number of limitations that should be borne in mind in interpreting the findings. Firstly, as this study was not pre‐registered, all analyses should be considered exploratory and interpreted with caution. As a result, future pre‐registered studies are needed to confirm these results. Additionally, participants were recruited through prolific, meaning that their diagnoses were self‐reported and not verified. It is also possible that individuals using prolific may be a more highly motivated and research‐engaged group, limiting the generalizability of our findings. Consequently, it may be beneficial for future studies to recruit from clinical settings, which allow for verification of participant's diagnosis and provide access to more representative samples. Participants in this study also had a wide range of different health conditions, some of which were rheumatic and not autoimmune (e.g., osteoarthritis) and some of which were both rheumatic and autoimmune (e.g., rheumatoid arthritis). It may be that associations differ across different illnesses, although research to date has suggested that the relationships are more consistent than inconsistent across even more disparate illnesses than sampled here (Sharpe, Richmond, et al., 2023). As such, we think that the broad nature of illnesses can be seen as a strength and that add to the generalizability of our results. While the broad range of illnesses may aid generalizability of the findings, our sample was nevertheless a well‐educated, largely white sample and therefore is unlikely to be generalizable in other ways. Moreover, most of the analyses were based on cross‐sectional relationships, whereby it is impossible to interpret the direction of causality. Therefore, while we can say that death anxiety appears important for persistent FoP, as death anxiety and FoP were highly correlated at baseline, it is possible that this finding could be the product of both constructs remaining elevated over time. Ultimately, future studies utilizing repeated measures designs and cross‐lagged modelling are needed to tease apart any potential causal relationship over time.

## CONCLUSIONS

Our results indicate that FoP and death anxiety may be much neglected but important constructs for people with rheumatic disorders. Based on clinical cut‐offs established for people with cancer, 73% of the participants with chronic conditions scored in the clinical range for FoP. While psychosocial outcomes were worse in those with clinically significant FoP than those without, it was death anxiety affect and beliefs that were associated with higher scores on FoP. Moreover, death anxiety continued to predict independent variance in FoP when controlling for all other psychosocial outcomes, and 3 months later. Although HRQoL outcomes appeared to be robust to independent impacts of FoP and death anxiety, greater research is needed to understand the possibly complex interplay between FoP, death anxiety and other important outcomes – such as healthcare behaviours.

## AUTHOR CONTRIBUTIONS

**Bethany Richmond:** Conceptualization; investigation; writing – original draft; methodology; validation; writing – review and editing; formal analysis; data curation; project administration. **Louise Sharpe:** Conceptualization; data curation; formal analysis; investigation; methodology; project administration; supervision; resources; validation; writing – review and editing. **Jack B. Boyse:** Conceptualization; data curation; formal analysis; investigation; writing – review and editing. **Joanne Shaw:** Conceptualization; supervision; writing – review and editing. **Rachel E. Menzies:** Conceptualization; data curation; investigation; methodology; project administration; supervision; validation; writing – review and editing.

## FUNDING INFORMATION

This study was supported by a generous bequest from the Dorothy Reavley Tinsley Trust.

## CONFLICT OF INTEREST STATEMENT

The authors declare no conflicts of interest.

## Data Availability

The data that support the findings of this study are available from the corresponding author upon reasonable request.

## References

[bjhp70069-bib-0001] Arch, J. J. , Slivjak, E. T. , & Finkelstein, L. B. (2024). A novel intervention to reduce fear of progression and trauma symptoms in advanced cancer using written exposure to worst‐case scenarios. Journal of Palliative Medicine, 27(8), 1009–1017. 10.1089/jpm.2023.0658 38579139

[bjhp70069-bib-0002] Berg, P. , Book, K. , Dinkel, A. , Henrich, G. , Marten‐Mittag, B. , Mertens, D. , Ossner, C. , Volmer, S. , & Herschbach, P. (2011). Fear of progression in chronic diseases. Psychotherapie, Psychosomatik, Medizinische Psychologie, 61(1), 32–37. 10.1055/s-0030-1267927 21120791

[bjhp70069-bib-0003] Boyse, J. B. , Sharpe, L. , Richmond, B. , Dear, B. , Dudeney, J. , Sesel, A. L. , & Menzies, R. E. (2024). Benign or painful? The interpretation of pain and fear of progression in rheumatoid arthritis. Pain, 165(4), 838–847. 10.1097/j.pain.0000000000003081 37889599

[bjhp70069-bib-0004] Cho, S. , & Cho, O. H. (2022). Depression and quality of life in older adults with pneumoconiosis: The mediating role of death anxiety. Geriatric Nursing, 44, 215–220. 10.1016/j.gerinurse.2022.02.018 35231755

[bjhp70069-bib-0005] Curran, L. , Sharpe, L. , MacCann, C. , & Butow, P. (2020). Testing a model of fear of cancer recurrence or progression: The central role of intrusions, death anxiety and threat appraisal. Journal of Behavioral Medicine, 43(2), 225–236. 10.1007/s10865-019-00129-x 31907743

[bjhp70069-bib-0006] Fardell, J. E. , Thewes, B. , Turner, J. , Gilchrist, J. , Sharpe, L. , Smith, A. , Girgis, A. , & Butow, P. (2016). Fear of cancer recurrence: A theoretical review and novel cognitive processing formulation. Journal of Cancer Survivorship, 10(4), 663–673. 10.1007/s11764-015-0512-5 26782171

[bjhp70069-bib-0007] Faul, F. , Erdfelder, E. , Buchner, A. , & Lang, A.‐G. (2009). Statistical power analyses using G*power 3.1: Tests for correlation and regression analyses. Behavior Research Methods, 41(4), 1149–1160. 10.3758/BRM.41.4.1149 19897823

[bjhp70069-bib-0008] Goldblatt, F. , & O'Neill, S. G. (2013). Clinical aspects of autoimmune rheumatic diseases. Lancet, 382(9894), 797–808. 10.1016/S0140-6736(13)61499-3 23993190

[bjhp70069-bib-0009] Goldenberg, J. L. , & Arndt, J. (2008). The implications of death for health: A terror management health model for behavioral health promotion. Psychological Review, 115(4), 1032–1053. 10.1037/a0013326 18954213

[bjhp70069-bib-0010] Gore, M. , Brandenburg, N. A. , Dukes, E. , Hoffman, D. L. , Tai, K. S. , & Stacey, B. (2005). Pain severity in diabetic peripheral neuropathy is associated with patient functioning, symptom levels of anxiety and depression, and sleep. Journal of Pain and Symptom Management, 30(4), 374–385. 10.1016/j.jpainsymman.2005.04.009 16256902

[bjhp70069-bib-0011] Hay, G. , Korwisi, B. , Rief, W. , Smith, B. H. , Treede, R. D. , & Barke, A. (2022). Pain severity ratings in the 11th revision of the international classification of diseases: A versatile tool for rapid assessment. Pain, 163(12), 2421–2429. 10.1097/j.pain.0000000000002640 35316821

[bjhp70069-bib-0012] Herschbach, P. , Berg, P. , Dankert, A. , Duran, G. , Engst‐Hastreiter, U. , Waadt, S. , Keller, M. , Ukat, R. , & Henrich, G. (2005). Fear of progression in chronic diseases: Psychometric properties of the fear of progression questionnaire. Journal of Psychosomatic Research, 58(6), 505–511. 10.1016/j.jpsychores.2005.02.007 16125517

[bjhp70069-bib-0013] Herschbach, P. , Berg, P. , Waadt, S. , Duran, G. , Engst‐Hastreiter, U. , Henrich, G. , Book, K. , & Dinkel, A. (2009). Group psychotherapy of dysfunctional fear of progression in patients with chronic arthritis or cancer. Psychotherapy and Psychosomatics, 79(1), 31–38. 10.1159/000254903 19887889

[bjhp70069-bib-0014] Iverach, L. , Menzies, R. G. , & Menzies, R. E. (2014). Death anxiety and its role in psychopathology: Reviewing the status of a transdiagnostic construct. Clinical Psychology Review, 34(7), 580–593. 10.1016/j.cpr.2014.09.002 25306232

[bjhp70069-bib-0015] Jenkinson, C. , Layte, R. , Jenkinson, D. , Lawrence, K. , Petersen, S. , Paice, C. , & Stradling, J. (1997). A shorter form health survey: Can the SF‐12 replicate results from the SF‐36 in longitudinal studies? Journal of Public Health Medicine, 19(2), 179–186. 10.1093/oxfordjournals.pubmed.a024606 9243433

[bjhp70069-bib-0016] Korwisi, B. , Hay, G. , Attal, N. , Aziz, Q. , Bennett, M. I. , Benoliel, R. , Cohen, M. , Evers, S. , Giamberardino, M. A. , Kaasa, S. , Kosek, E. , Lavand'homme, P. , Nicholas, M. , Perrot, S. , Schug, S. , Smith, B. H. , Svensson, P. , Vlaeyen, J. W. S. , Wang, S. J. , … Barke, A. (2021). Classification algorithm for the international classification of Diseases‐11 chronic pain classification: Development and results from a preliminary pilot evaluation. Pain, 162(7), 2087–2096. 10.1097/j.pain.0000000000002208 33492033

[bjhp70069-bib-0017] Liu, Q. W. , Qin, T. , Hu, B. , Zhao, Y. L. , & Zhu, X. L. (2021). Relationship between illness perception, fear of progression and quality of life in interstitial lung disease patients: A cross‐sectional study. Journal of Clinical Nursing, 30(23–24), 3493–3505. 10.1111/jocn.15852 33998090

[bjhp70069-bib-0018] Lovibond, S. H. , & Lovibond, P. (1996). Depression anxiety stress scales (2nd ed.). Psychology Foundation.

[bjhp70069-bib-0019] Luigjes‐Huizer, Y. L. , Tauber, N. M. , Humphris, G. , Kasparian, N. A. , Lam, W. W. T. , Lebel, S. , Simard, S. , Smith, A. B. , Zachariae, R. , Afiyanti, Y. , Bell, K. J. L. , Custers, J. A. E. , de Wit, N. J. , Fisher, P. L. , Galica, J. , Garland, S. N. , Helsper, C. W. , Jeppesen, M. M. , Liu, J. , … van der Lee, M. L. (2022). What is the prevalence of fear of cancer recurrence in cancer survivors and patients? A systematic review and individual participant data meta‐analysis. Psycho‐Oncology, 31(6), 879–892. 10.1002/pon.5921 35388525 PMC9321869

[bjhp70069-bib-0020] Matcham, F. , Rayner, L. , Steer, S. , & Hotopf, M. (2013). The prevalence of depression in rheumatoid arthritis: A systematic review and meta‐analysis. Rheumatology (Oxford, England), 52(12), 2136–2148. 10.1093/rheumatology/ket169 24003249 PMC3828510

[bjhp70069-bib-0021] Mau, W. , Listing, J. , Huscher, D. , Zeidler, H. , & Zink, A. (2005). Employment across chronic inflammatory rheumatic diseases and comparison with the general population. The Journal of Rheumatology, 32(4), 721–728. 15801031

[bjhp70069-bib-0022] Menzies, R. E. , Richmond, B. , Sharpe, L. , Skeggs, A. , Liu, J. , & Coutts‐Bain, D. (2024). The ‘revolving door’ of mental illness: A meta‐analysis and systematic review of current versus lifetime rates of psychological disorders. British Journal of Clinical Psychology, 63(2), 178–196. 10.1111/bjc.12453 38197576

[bjhp70069-bib-0023] Menzies, R. E. , Sharpe, L. , & Dar‐Nimrod, I. (2019). The relationship between death anxiety and severity of mental illnesses. The British Journal of Clinical Psychology, 58(4), 452–467. 10.1111/bjc.12229 31318066

[bjhp70069-bib-0024] Menzies, R. E. , Sharpe, L. , & Dar‐Nimrod, I. (2022). The development and validation of the death anxiety beliefs and Behaviours scale. The British Journal of Clinical Psychology, 61(4), 1169–1187. 10.1111/bjc.12387 35938594 PMC9804815

[bjhp70069-bib-0025] Menzies, R. E. , Zuccala, M. , Sharpe, L. , & Dar‐Nimrod, I. (2018). The effects of psychosocial interventions on death anxiety: A meta‐analysis and systematic review of randomised controlled trials. Journal of Anxiety Disorders, 59, 64–73. 10.1016/j.janxdis.2018.09.004 30308474

[bjhp70069-bib-0026] Michalski, S. C. , Sharpe, L. , Boyse, J. B. , Shaw, J. , & Menzies, R. E. (2024). The role of pain and interpretation bias in fear of disease progression in people with diabetes. The Journal of Pain, 25(11), 104638. 10.1016/j.jpain.2024.104638 39025285

[bjhp70069-bib-0027] Michaud, K. , & Wolfe, F. (2007). Comorbidities in rheumatoid arthritis. Best Practice & Research. Clinical Rheumatology, 21(5), 885–906. 10.1016/j.berh.2007.06.002 17870034

[bjhp70069-bib-0028] Moran, C. , Tomei, C. , Lefebvre, M. , Harris, C. , Maheu, C. , & Lebel, S. (2017). An exploratory study of the worst‐case scenario exercise as an exposure treatment for fear of cancer recurrence. Supportive Care in Cancer, 25(5), 1373–1375. 10.1007/s00520-017-3600-4 28150044

[bjhp70069-bib-0029] Oliver, J. E. , & Silman, A. J. (2009). Why are women predisposed to autoimmune rheumatic diseases? Arthritis Research & Therapy, 11(5), 252. 10.1186/ar2825 19863777 PMC2787267

[bjhp70069-bib-0030] Pisetsky, D. S. (2023). Pathogenesis of autoimmune disease. Nature Reviews. Nephrology, 19(8), 509–524. 10.1038/s41581-023-00720-1 37165096 PMC10171171

[bjhp70069-bib-0031] Pradhan, P. , Sharpe, L. , Butow, P. , Coutts‐Bain, D. , & Heathcote, L. C. (2022). Does interpretation bias moderate the relationship between pain and fear of cancer recurrence? Health Psychology, 41(11), 874–883. 10.1037/hea0001217 36074599

[bjhp70069-bib-0032] Pradhan, P. , Sharpe, L. , Butow, P. , & Russell, H. (2021). The role of interpretation biases and symptom burden in fear of cancer recurrence/progression among ovarian cancer survivors. Psycho‐Oncology, 30(11), 1948–1956. 10.1002/pon.5748 34106498

[bjhp70069-bib-0033] Restivo, V. , Candiloro, S. , Daidone, M. , Norrito, R. , Cataldi, M. , Minutolo, G. , Caracci, F. , Fasano, S. , Ciccia, F. , Casuccio, A. , & Tuttolomondo, A. (2022). Systematic review and meta‐analysis of cardiovascular risk in rheumatological disease: Symptomatic and non‐symptomatic events in rheumatoid arthritis and systemic lupus erythematosus. Autoimmunity Reviews, 21(1), 102925. 10.1016/j.autrev.2021.102925 34454117

[bjhp70069-bib-0034] Sangha, O. (2000). Epidemiology of rheumatic diseases. Rheumatology, 39(2), 3–12. 10.1093/rheumatology/39.suppl_2.3 11276800

[bjhp70069-bib-0035] Sendlbeck, M. , Araujo, E. G. , Schett, G. , & Englbrecht, M. (2015). Psychometric properties of three single‐item pain scales in patients with rheumatoid arthritis seen during routine clinical care: A comparative perspective on construct validity, reproducibility and internal responsiveness. RMD Open, 1(1), e000140. 10.1136/rmdopen-2015-000140 26719815 PMC4692050

[bjhp70069-bib-0036] Sharpe, L. , Bisby, M. A. , Menzies, R. E. , Richmond, B. , Todd, J. , Sesel, A. L. , & Dear, B. F. (2025). A tale of two treatments: A randomised controlled trial of mindfulness or cognitive behaviour therapy delivered online for people with rheumatoid arthritis. Psychotherapy and Psychosomatics, 94(2), 89–100. 10.1159/000542489 39827846

[bjhp70069-bib-0037] Sharpe, L. , Menzies, R. E. , Boyse, J. , Bisby, M. A. , Richmond, B. , Todd, J. , Sesel, A. L. , & Dear, B. F. (2025). Mediators and moderators of two online interventions for managing pain, fear of progression and functional ability in rheumatoid arthritis. Behaviour Research and Therapy, 185, 104676. 10.1016/j.brat.2024.104676 39742659

[bjhp70069-bib-0038] Sharpe, L. , Menzies, R. E. , Richmond, B. , Todd, J. , MacCann, C. , & Shaw, J. (2024). The development and validation of the worries about recurrence or progression scale (WARPS). British Journal of Health Psychology, 29(2), 454–467. 10.1111/bjhp.12707 38040446

[bjhp70069-bib-0039] Sharpe, L. , Michalowski, M. , Richmond, B. , Menzies, R. E. , & Shaw, J. (2023). Fear of progression in chronic illnesses other than cancer: A systematic review and meta‐analysis of a transdiagnostic construct. Health Psychology Review, 17(2), 301–320. 10.1080/17437199.2022.2039744 35132937

[bjhp70069-bib-0040] Sharpe, L. , Richmond, B. , Todd, J. , Dudeney, J. , Dear, B. F. , Szabo, M. , Sesel, A. L. , Forrester, M. , & Menzies, R. E. (2023c). A cross‐sectional study of existential concerns and fear of progression in people with rheumatoid arthritis. Journal of Psychosomatic Research, 175, 111514. 10.1016/j.jpsychores.2023.111514 37883892

[bjhp70069-bib-0041] Sharpe, L. , Richmond, B. , Todd, J. , Dudeney, J. , Dear, B. F. , Szabo, M. , Sesel, A.‐L. , Forrester, M. , & Menzies, R. E. (2023b). A cross‐sectional study of existential concerns and fear of progression in people with rheumatoid arthritis. Journal of Psychosomatic Research, 175, 111514. 37883892 10.1016/j.jpsychores.2023.111514

[bjhp70069-bib-0042] Sherman, D. W. , Norman, R. , & McSherry, C. B. (2010). A comparison of death anxiety and quality of life of patients with advanced cancer or AIDS and their family caregivers. Journal of the Association of Nurses in AIDS Care, 21(2), 99–112. 10.1016/j.jana.2009.07.007 20006525

[bjhp70069-bib-0043] Shi, Y. , Li, M. , Liu, L. , Wang, Z. , Wang, Y. , Zhao, J. , Wang, Q. , Tian, X. , Li, M. , & Zeng, X. (2021). Relationship between disease activity, organ damage and health‐related quality of life in patients with systemic lupus erythematosus: A systemic review and meta‐analysis. Autoimmunity Reviews, 20(1), 102691. 10.1016/j.autrev.2020.102691 33190803

[bjhp70069-bib-0044] Shrestha, S. , Sapkota, S. , Teoh, S. L. , KC, B. , Paudyal, V. , Lee, S. W. H. , & Gan, S. H. (2024). Comprehensive assessment of pain characteristics, quality of life, and pain management in cancer patients: A multi‐center cross‐sectional study. Quality of Life Research, 33(10), 2755–2771. 10.1007/s11136-024-03725-w 39105961 PMC11452497

[bjhp70069-bib-0045] Simard, S. , Thewes, B. , Humphris, G. , Dixon, M. , Hayden, C. , Mireskandari, S. , & Ozakinci, G. (2013). Fear of cancer recurrence in adult cancer survivors: A systematic review of quantitative studies. Journal of Cancer Survivorship, 7(3), 300–322. 10.1007/s11764-013-0272-z 23475398

[bjhp70069-bib-0046] Simonelli, L. E. , Siegel, S. D. , & Duffy, N. M. (2017). Fear of cancer recurrence: A theoretical review and its relevance for clinical presentation and management. Psycho‐Oncology, 26(10), 1444–1454. 10.1002/pon.4168 27246348

[bjhp70069-bib-0047] Smith, M. , Sharpe, L. , Winiarski, N. , & Shaw, J. (2024). The worries about recurrence or progression scale in cancer (WARPS‐C): A valid and reliable measure to screen for fear of cancer recurrence. Psycho‐Oncology, 33(12), e70055. 10.1002/pon.70055 39706806

[bjhp70069-bib-0048] Sokol, L. L. , Troost, J. P. , Bega, D. , Paulsen, J. S. , Kluger, B. M. , Applebaum, A. J. , Frank, S. , Nance, M. A. , Anderson, K. E. , Perlmutter, J. S. , Depp, C. A. , Grafman, J. , Cella, D. , & Carlozzi, N. E. (2023). Death anxiety in Huntington disease: Longitudinal heath‐related quality‐of‐life outcomes. Journal of Palliative Medicine, 26(7), 907–914. 10.1089/jpm.2022.0160 36607769 PMC10316526

[bjhp70069-bib-0049] Szymczak, H. , Brandstetter, S. , Ehrenstein, B. , Steinmann, M. , & Apfelbacher, C. (2025). Predictors for health‐related quality of life in patients with rheumatoid arthritis: A longitudinal study. Rheumatology Advances in Practice, 9(4), rkaf116. 10.1093/rap/rkaf116 41221504 PMC12597878

[bjhp70069-bib-0050] Tan, J. A. , Dehghan, N. , Chen, W. , Xie, H. , Esdaile, J. M. , & Avina‐Zubieta, J. A. (2017). Mortality in ANCA‐associated vasculitis: Ameta‐analysis of observational studies. Annals of the Rheumatic Diseases, 76(9), 1566–1574. 10.1136/annrheumdis-2016-210942 28468793

[bjhp70069-bib-0051] Thewes, B. , Butow, P. , Bell, M. L. , Beith, J. , Stuart‐Harris, R. , Grossi, M. , Capp, A. , Dalley, D. , & FCR Study Advisory Committee . (2012). Fear of cancer recurrence in young women with a history of early‐stage breast cancer: A cross‐sectional study of prevalence and association with health behaviours. Supportive Care in Cancer, 20(11), 2651–2659. 10.1007/s00520-011-1371-x 22328003

[bjhp70069-bib-0052] Thong, I. S. K. , Jensen, M. P. , Miró, J. , & Tan, G. (2018). The validity of pain intensity measures: What do the NRS, VAS, VRS, and FPS‐R measure? Scandinavian Journal of Pain, 18(1), 99–107. 10.1515/sjpain-2018-0012 29794282

[bjhp70069-bib-0053] Toledano, E. , Candelas, G. , Rosales, Z. , Prada, C. M. , León, L. , Abásolo, L. , Loza, E. , Carmona, L. , Tobías, A. , & Jover, J. Á. (2012). A meta‐analysis of mortality in rheumatic diseases. Reumatología Clínica, 8(6), 334–341. 10.1016/j.reuma.2012.05.006 22789463

[bjhp70069-bib-0054] van der Zee‐Neuen, A. , Putrik, P. , Ramiro, S. , Keszei, A. P. , Hmamouchi, I. , Dougados, M. , & Boonen, A. (2017). Large country differences in work outcomes in patients with RA – An analysis in the multinational study COMORA. Arthritis Research & Therapy, 19(1), 216. 10.1186/s13075-017-1421-y 28962581 PMC5622486

[bjhp70069-bib-0055] Wang, Y. , Yu, Q. , Zeng, Z. , Yuan, R. , Wang, R. , Chen, J. , Zhou, H. , & Tang, J. (2022). Predictors of fear of diabetes progression: A multi‐center cross‐sectional study for patients self‐management and healthcare professions education. Frontiers in Public Health, 10, 910145. 10.3389/fpubh.2022.910145 36600932 PMC9806215

[bjhp70069-bib-0056] Ware, J., Jr. , Kosinski, M. , & Keller, S. D. (1996). A 12‐item short‐form health survey: Construction of scales and preliminary tests of reliability and validity. Medical Care, 34(3), 220–233. 10.1097/00005650-199603000-00003 8628042

[bjhp70069-bib-0057] Williams, J. T. W. , Pearce, A. , & Smith, A' . (2021). A systematic review of fear of cancer recurrence related healthcare use and intervention cost‐effectiveness. Psycho‐Oncology, 30(8), 1185–1195. 10.1002/pon.5673 33880822

[bjhp70069-bib-0058] Zhang, L. , Cai, P. , & Zhu, W. (2020). Depression has an impact on disease activity and health‐related quality of life in rheumatoid arthritis: A systematic review and meta‐analysis. International Journal of Rheumatic Diseases, 23(3), 285–293. 10.1111/1756-185x.13774 31858731

[bjhp70069-bib-0059] Zhang, L. , Fu, T. , Yin, R. , Zhang, Q. , & Shen, B. (2017). Prevalence of depression and anxiety in systemic lupus erythematosus: A systematic review and meta‐analysis. BMC Psychiatry, 17(1), 70. 10.1186/s12888-017-1234-1 28196529 PMC5310017

[bjhp70069-bib-0060] Zusman, E. Z. , Howren, A. M. , Park, J. Y. E. , Dutz, J. , & De Vera, M. A. (2020). Epidemiology of depression and anxiety in patients with psoriatic arthritis: A systematic review and meta‐analysis. Seminars in Arthritis and Rheumatism, 50(6), 1481–1488. 10.1016/j.semarthrit.2020.02.001 32178850

